# Medical Opinion and Movement

**Published:** 1908-01-25

**Authors:** 


### Medical Opinion and Movement.

After working at the subject for the past two years Dr. Simon Flexner, of the Rockefeller Institute, has succeeded in producing a serum which offers promising results in the treatment of cerebro-spinal meningitis. The serum is prepared by inoculating horses with the cerebro-spinal fluid taken from patients afflicted with the disease. It is claimed that the serum has now proved successful in over 60 cases, and that in no instance in which it was employed was it ineffectual. It is admitted that the cases in which this serum has been tried may not have been so virulent as those occurring at the height of the epidemic which began in New York in 1905. Nevertheless, the disease has remained sufficiently severe to destroy an average of eight lives each week in the city during the past three months. Further evidence of the efficacy of the serum will be awaited with interest.

There is undoubtedly a growing disposition on the part of public bodies to give certain appointments to medical women, largely on the ground that they will be willing to perform the duties at a smaller remuneration than will medical men. In the near future a good many appointments will probably be made in connection with the administration of the new Education Act, and there are already indications that local authorities will endeavour to run the Act on economical lines by offering these appointments to medical women at salaries which would be refused by male members of the profession. It is satisfactory, therefore, in view of these contingencies, that the Association of Registered Medical Women has passed a resolution at a recent meeting to the effect that women who have adopted the profession of medicine are entitled to the same remuneration as their male *confrères*, and that ethically, as well as in their own interest, they should refuse to accept salaries lower than those offered to men doing the same kind of work. By a further resolution a Vigilance Committee has been formed to watch advertisements of vacancies offered to women candidates, and to warn medical women not to apply for posts for which an inadequate salary is offered. On more than one occasion medical women have shown a proper public spirit, and have sacrificed their own immediate interests to the general good of the profession, and there is no doubt that by refusing to accept a lower grade of remuneration, they will serve their own welfare by securing a proper recognition of the value of their services.

A good deal of attention and research have been given recently to the determination of the nature of the toxicity of therapeutic sera, which occasionally manifests itself in the form of so-called "serum disease" after inoculation. The condition consists in the appearance of an exanthem, generally urticarial in nature, at the site of injection eight to twelve days after inoculation, accompanied by an alteration of temperature and local glandular enlargement. The rash tends to spread rapidly over the body. There is generally considerable œdema and occasionally slight albuminuria. Convalescence occurs on the third or fourth day. A fatal issue is extremely rare. So far the nature of the toxin or sensitizing substance has not been ascertained, but it appears to be specific both in quantity and character, and the degree of toxicity of any serum can be determined by the inoculation of guinea pigs which have been sensitized with a previous dose of serum. Besredka, in discussing the question, considers that sera intended for therapeutic use should be tested systematically for toxic as well as antitoxic properties in this way. No serum should be used if its degree of toxicity is above the average—that is if death or severe disturbance is produced in a sensitized guinea pig by a dose not exceeding $\frac{1}{20}$ c.c.m. Horse sera of different origins often vary greatly in toxicity, but it is clearly established that a serum is most toxic when first drawn, and that the toxicity rapidly diminishes during the first ten days. Afterwards, during the following six weeks, the fall in toxicity is much slower. After two months little or no change takes place on keeping. It appears from these facts that no serum should be considered sufficiently free from toxicity until it is at least two months old.

The question of typhoid infection by "chronic typhoid carriers" has not hitherto received much consideration in this country, but it has been the subject of investigation by bacteriologists in Germany for the past two years. In Strassburg outbreaks of the fever were definitely traced in one case to a female baker, and in another instance to a woman engaged in the milk trade, whose fæces were found to contain the typhoid bacilli in large quantities. In a similar way a succession of typhoid epidemics in an asylum were found on careful investigation by Nieter and Liefmann to be due to "typhoid carriers" among the inmates. Similar results have now been obtained in an asylum in Scotland by Drs. Alex. and J. C. G. Ledingham. The asylum has been visited by a succession of outbreaks of typhoid fever since 1893. Altogether thirty-one cases have occurred, twenty-four being females. Nine cases have proved fatal. Repeated analyses of the water supply failed to throw any light on the cause of these outbreaks. Sanitary arrangements were as complete as possible, and the dairy was ascertained to be in excellent order. It was therefore eventually decided to investigate the possibility of "typhoid carriers" as the cause of these outbreaks. By bacteriological examination of the stools of fifty females, a typhoid carrier was isolated with an enormous number of typhoid bacilli in the stools. After the isolation of this patient a further outbreak took place in the small asylum, and consequently a further investigation was made, which resulted in the isolation of two more typhoid carriers. These results are highly interesting and important, and open up a new line of investigation in typhoid epidemics of obscure origin. The bacilli vegetate in the gall bladder, from which they are intermittently ejected into the intestine. Intestinal antiseptics seem to be of little avail.

